# Multi-Scale Attention 3D Convolutional Network for Multimodal Gesture Recognition

**DOI:** 10.3390/s22062405

**Published:** 2022-03-21

**Authors:** Huizhou Chen, Yunan Li, Huijuan Fang, Wentian Xin, Zixiang Lu, Qiguang Miao

**Affiliations:** 1 School of Computer Science and Technology, Xidian University, Xi’an 710071, China; huizhouchen@stu.xidian.edu.cn (H.C.); wtxin@stu.xidian.edu.cn (W.X.); zxlu@xidian.edu.cn (Z.L.); 2 Xiaomi Communications, Beijing 100085, China; hjfang95@163.com

**Keywords:** gesture recognition, multi-scale attention, multimodal data

## Abstract

Gesture recognition is an important direction in computer vision research. Information from the hands is crucial in this task. However, current methods consistently achieve attention on hand regions based on estimated keypoints, which will significantly increase both time and complexity, and may lose position information of the hand due to wrong keypoint estimations. Moreover, for dynamic gesture recognition, it is not enough to consider only the attention in the spatial dimension. This paper proposes a multi-scale attention 3D convolutional network for gesture recognition, with a fusion of multimodal data. The proposed network achieves attention mechanisms both locally and globally. The local attention leverages the hand information extracted by the hand detector to focus on the hand region, and reduces the interference of gesture-irrelevant factors. Global attention is achieved in both the human-posture context and the channel context through a dual spatiotemporal attention module. Furthermore, to make full use of the differences between different modalities of data, we designed a multimodal fusion scheme to fuse the features of RGB and depth data. The proposed method is evaluated using the Chalearn LAP Isolated Gesture Dataset and the Briareo Dataset. Experiments on these two datasets prove the effectiveness of our network and show it outperforms many state-of-the-art methods.

## 1. Introduction

The gesture is a natural form of human communication, and can convey rich semantic information through hand position, shape, pointing, etc. Therefore, gesture recognition is an important direction in computer vision research, and has been widely used in virtual reality, smart home, and other fields.

In recent years, there has been a wealth of research on gesture recognition. The position and shape of the performer’s hands are crucial in gesture recognition tasks, so it is natural to make use of hand information when establishing an attention mechanism to improve the network’s performance. However, many current methods build local attention mechanisms using a heatmap generated from extracted key points on the hands [1,2,3]. These methods require specialized networks to obtain the skeletal information regarding the human body, which significantly increases the cost of learning, and blurs the position information of the hands in the process of generating the heatmap. Therefore, it is a more effective method to establish local attention by directly obtaining hand information through hand detection of RGB data. In addition, many gesture recognition methods only consider attention mechanisms in the spatial domain. However, for dynamic gesture recognition, attention of the spatial and temporal domains should be considered comprehensively since the temporal information also benefits the recognition of hand motions.

**Figure 1:**
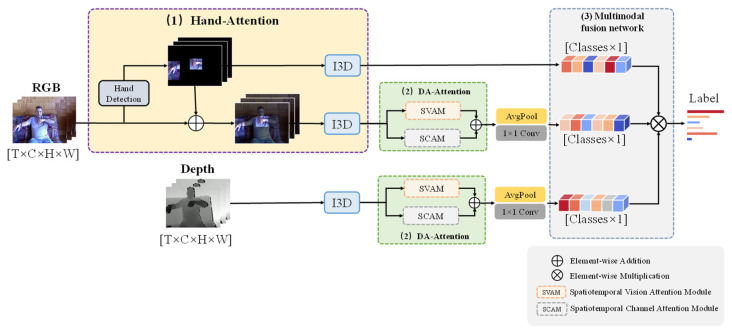
Our pipeline of the proposed network. This network takes I3D [4] as the backbone and consists of three parts: (**1**) A local attention module to enhance the network’s attention to the hand region. (**2**) A dual spatiotemporal attention module to extract global spatiotemporal posture context information. (**3**) A multimodal fusion network for fusing different modality features.

In this article, we propose a multi-scale attention 3D convolutional network (MSA-3D) for large-scale dynamic gesture recognition tasks. The pipeline of the proposed approach is shown in Figure 1. Firstly, considering that dynamic gesture recognition has a strong temporal dependence, the network takes the I3D [4] network as the baseline to extract the spatiotemporal features in different receptive fields, and learns the motion information of the gesture. Secondly, to explicitly highlight the information related to the gesture, we propose a multi-scale attention scheme. From the local view, we employ a hand detector to mark the hand region and highlight it in each frame. This makes the network pay more attention to the performer’s hand, and avoid missing parts of the hand due to imprecise estimation of hand keypoints. For the global view, we achieve attention in two aspects. We designed a dual spatiotemporal attention module by combining spatiotemporal vision and spatiotemporal channel attention mechanisms to extract the human-posture context information in the global spatiotemporal dimension. Moreover, in the large-scale dynamic gesture recognition task, the single modality data are not enough to fully present features. Specifically, RGB data can represent rich texture features, but using RGB data alone for gesture recognition can often face interference from factors such as illumination changes and shading. In comparison, depth data are only related to the distance from the objects to the sensors. To make comprehensive use of the different modalities of data, we designed a multimodal fusion network to extract and fuse the features of both RGB data and depth data, making the features more robust.

Our contributions are summarized below.

We proposed a multi-scale attention 3D convolution network for gesture recognition. We used the local attention mechanism with the hand detector to help the network pay attention to hand information so as to reduce the influence of factors irrelevant to the gesture recognition task.

For global attention, we designed a dual spatiotemporal attention scheme that combines the spatiotemporal vision and spatiotemporal channel attention schemes to extract global spatiotemporal posture context information.

To make full use of the advantages of RGB data and depth data, we designed a multimodal fusion network for fusing features of different modalities of data.

## 2. Related Works

Gesture recognition is one of the main research fields in computer vision in recent decades. Early gesture recognition methods were generally based on handcrafted features [5,6,7,8,9]. With the rapid development of deep learning, many gesture recognition methods based on the deep neural network have appeared. Firstly, for action recognition, Simonyan and Ziserman [10] proposed using two-stream convolutional networks, derived from 2D convolutional neural networks. Later, some researchers used LSTM and its variants to capture the temporal information of gestures. Donahue et al. [11] captured spatial information from a convolutional neural network, acquired temporal information from a long short-term memory (LSTM), and combined it to form a long-term recurrent convolutional neural network. Furthermore, some researchers used a 3D convolutional neural network (3DCNN) for gesture recognition [4,12,13]. There are also some methods that used other modality data, besides RGB-D. For example, Miao et al. [14] used a 3DCNN to learn spatiotemporal features from RGB, depth, and optical flow data, and fused these features by using canonical correlation analysis (CCA). Li et al. [15] and Duan et al. [16] leveraged saliency data to improve the performance of the model. In addition, some methods do not use the original RGB-D features. Wang et al. [17] use the dynamic images instead of the raw RGB-D data as the input for gesture recognition.

Attention mechanisms have been widely used in computer vision tasks. Background interference and changes in the performer’s clothing are still obstacles to improving gesture recognition accuracy. Some methods use attention mechanisms that guide the network to focus on the gesture itself. Liu et al. [18] used fast-RCNN [19] as a hand detector and highlighted the corresponding area. Narayana et al. [20] used a focus of the attention network to extract global data and local data of left and right hands, respectively. Li et al. [15] and Miao et al. [14] used optical flow data to guide the network to learn the motion information between frames. However, for dynamic gesture recognition tasks, it is not enough to consider only the spatial domain and the temporal domain. The dependence between spatiotemporal and channel dimensions should also be considered comprehensively.

## 3. Approach

As shown in Figure 1, the proposed network can be divided into three parts: (1) a local attention module, (2) a dual spatiotemporal attention module (DSAM), and (3) a multimodal fusion network. The first two parts form the multi-scale attention mechanism. The task of the local attention module is to detect the performer’s hand position information in the video through a hand detector. It then uses this information as the attention signal to increase the weight of the areas related to gesture recognition tasks, such as the hands and arms in the video. It can reduce the influence of the background, the performer’s facial expression, and other irrelevant factors. The dual spatiotemporal attention module contains the spatiotemporal vision attention module (SVAM) and spatiotemporal channel attention module (SCAM). The SVAM uses local features to extract global spatiotemporal context information, and SCAM is used to extract the dependencies between different channels. As for the multimodal fusion network, it is used to process data of two different modalities: RGB and depth. This network can extract more robust features by using the complementarity between different modality data.

### 3.1. Local Attention Module

As mentioned in Section 1, hand information plays a key role in gesture recognition. However, extracting only the performer’s hand information does not fully represent a gesture, as dynamic gestures are also related to information such as the direction and order of movement of the performer’s arms. We hope that the network can keep part of the general information while paying attention to the hand information in the gesture recognition task. Therefore, we propose a local attention module based on hand detection. As shown in Figure 1, this module consists of the main branch and a hand detection branch.

**Figure 2:**
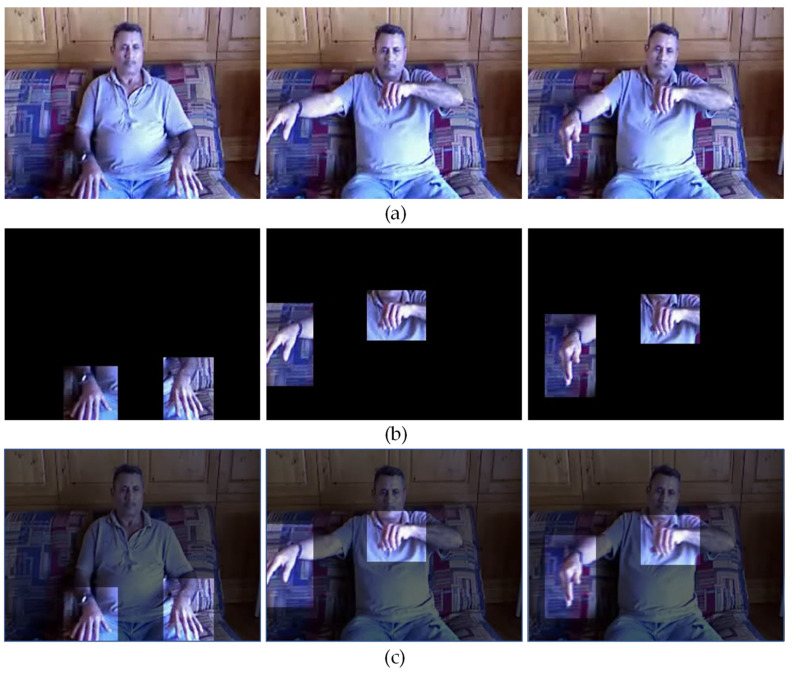
Sample of different data used in local attention: (**a**) raw RGB data; (**b**) hand data processed by hand detector; (**c**) fusion of raw RGB data and hand data.

The hand detecting branch uses YOLO v5 [21] as a hand detector to obtain the hand position and shape in RGB data, ignoring image information unrelated to the performer’s hand. Specifically, we trained it on the Oxford hand dataset [22] and performed hand detection on each frame of the video samples. For each frame, we only kept the information of the hand region and removed the rest of the background information. The RGB data processed by the hand detector are shown in Figure 2. Such processing made the network in this branch only consider the spatial characteristics of the hand area, which can effectively reduce the negative influence of factors such as background and clothing. At the same time, the main branch took the fusion of original data and hand data as it was input. This made the hand region in the original data more prominent, while retaining the overall information. The features obtained from this branch not only retain the spatial relationship of the original data, but also have a higher weight on the hand region.

### 3.2. Dual Spatiotemporal Attention Module

The position of a gesture in space and the proportion of a gesture to the image are different, and the convolution operation leads to the problem of the local receptive field. This makes it difficult to relate the features corresponding to the same hand region in the long temporal range to each other. Therefore, we designed a dual attention module based on [23] to capture remote context information in spatial and channel dimensions. However, in the task of dynamic gesture recognition, not only spatial features and channel features need to be considered in the spatial domain, but features of the temporal domain also need to be extracted. Hence, we extended the dual attention module based on the spatial domain to the temporal domain, and proposed a dual spatiotemporal attention module, as shown in Figure 1. This module consisted of a spatiotemporal vision attention module (SVAM) focusing on spatiotemporal similarity, and a spatiotemporal channel attention module (SCAM) focusing on the dependence between channels in spatiotemporal features. The features extracted from the I3D network were used as the inputs for SVAM and SCAM, respectively. After the reintegration of these two sub-modules, the features were first spatially embedded, then combined as the output of the dual spatiotemporal attention module.

**Figure 3:**
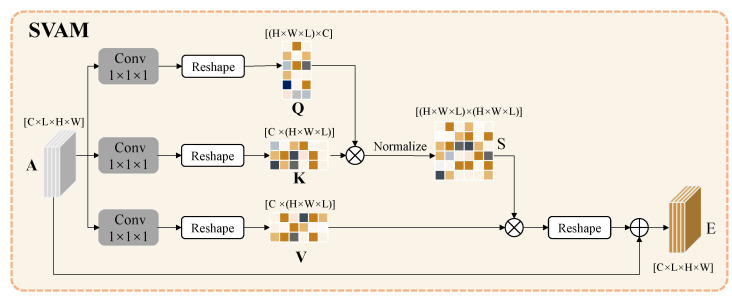
Architecture of SVAM. SVAM is designed for capturing the vision dependence in the spatiotemporal domain. This module takes the feature extracted by I3D as input. *Q*, *K*, and V correspond to query, key, and value matrices in the attention mechanism. The output feature is the result of the selective aggregation of features at all positions with the weighted sum of the input features.

As shown in Figure 3, the architecture of SVAM obtained a better feature representation by using local features to build global context information. Specifically, SVAM embedded the input feature $A\in R^{C\times L\times H\times W}$ through convolution and reshaped the operation to generate query, key and value matrices in the attention mechanism, corresponding to *Q*, *K* and V, respectively, in Figure 3. After that, the *Q* and *K* matrices were used for matrix multiplication to model the spatiotemporal relationships between any two spatiotemporal positions of the feature. Furthermore, the spatiotemporal attention weight distribution matrix *s* was generated after normalization using softmax. The calculation process of matrix *s* is shown in Equation (1).

(1) $$s_{ij}=\frac{\exp(Q_{i}\cdot K_{j})}{\sum_{j=1}^{N}\exp(Q_{i}\cdot K_{j})}$$

where $Q\in R^{N\times C}$, $K,V\in R^{C\times N}$, and N=H×W×L denote the matrices after embedding from *A*. Coefficients *H*, *W*, *L*, and *C* denote the height, width, length, and channel of the feature map. $s_{ij}$ is the element of the spatiotemporal attention weight distribution, matrix *s*, and denotes the similarity between position *i* and position *j*. The result of SVAM can be formulated as Equation (2).

(2) $$E_{i}=\alpha \sum_{j=1}^{N}(s_{ij}V_{i})+A_{i}$$

where *E* is the result of spatiotemporal vision attention. It is obtained by multiplying matrices *S* and *V*, then multiplying a learnable parameter α, and performing an element-wise sum operation with feature *A*. Each position in *E* is the result of the selective aggregation of features at all positions with the weighted sum of the original features. Thus, the feature of each position contains information of the global context.

**Figure 4:**
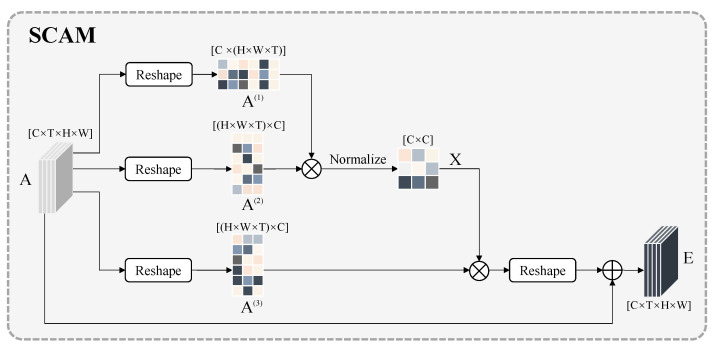
Architecture of SCAM. SCAM takes the feature extracted by I3D as input, which is designed for capturing the dependence between channels in the spatiotemporal domain. $A^{\left(1\right)},A^{\left(2\right)},A^{\left(3\right)}$ correspond to query, key, and value matrices in the attention mechanism. The output feature of each channel is the result of the selective aggregation of features on all channels, and the weighted sum of the input features.

Each channel feature map can be seen as a specific response to the current task in high-level semantics, and is correlated with different semantic responses. The spatiotemporal channel attention module explicitly models the interdependencies between channels and captures the remote context information in the channel dimension. The process of the channel attention module is similar to that of the spatiotemporal attention module, and its structure is shown in Figure 4. The input feature $A\in R^{C\times L\times H\times W}$ is reshaped into $A^{\left(1\right)}\in R^{C\times N}$, $A^{\left(2\right)},A^{\left(3\right)}\in R^{N\times C}$, and N=H×W×L. After that, as per Equations (3) and (4), the spatiotemporal channel attention weight distribution matrix $x\in R^{C\times C}$ is obtained by performing a matrix multiplication of A and $A^{\left(1\right)}$ which is normalized with softmax. After multiplying matrices x and $A^{\left(3\right)}$, the result is applied to $A^{\left(3\right)}$ and multiplied by a learnable parameter β, and, finally, summed element-wise with A.

(3) $$x_{ij}=\frac{\exp(A_{i}^{\left(1\right)}\cdot A_{j}^{\left(2\right)})}{\sum_{j=1}^{C}\exp(A_{i}^{\left(1\right)}\cdot A_{j}^{\left(2\right)})}$$

(4) $$E_{i}=\beta \sum_{j=1}^{C}(x_{ij}A_{j}^{\left(3\right)})+A_{i}$$

where $x_{ij}$ is the element of the spatiotemporal channel attention weight distribution matrix x, which denotes the influence of the *i*-th channel on the *j*-th channel. Coefficient Z denotes the result of the spatiotemporal channel attention module. Similar to SVAM, the feature map of each channel, after being processed by the spatiotemporal channel attention module, is the result of the selective aggregation of features on all channels, and the weighted sum of the original features. Therefore, the processed feature maps can capture the long-term semantics between channels.

### 3.3. Multimodal Fusion Module

Different modalities of data usually show consistency in some salient features and are complementary in some detailed features on the same object. For example, RGB data can better represent the texture features of objects, while depth data can better represent the distance information between objects and sensors. Therefore, this paper proposed using a multimodal fusion network to reasonably fuse multimodal information. This is so that the features extracted for gesture recognition would be more abundant and comprehensive, thereby improving the recognition accuracy.

This paper designed a multimodal fusion network based on the decision-level fusion method. As shown in Figure 1, we used the network branches with the local attention module and dual spatiotemporal attention module of RGB data to obtain the probability distribution. For depth data, the distribution was obtained using the branches with the dual spatiotemporal attention module. An element-wise multiplication operation was adopted for decision-level fusion, and the final probability result of the category was the product of the prediction distribution of each branch.

## 4. Experiments and Results

### 4.1. Dataset

To evaluate the performance of our proposed method, we conducted experiments on a large RGB-D gesture datasets: the Chalearn LAP IsoGD dataset and the Briareo dataset. 

ChaLearn LAP IsoGD dataset is referred to as the IsoGD dataset, which was released by Wan et al. [24] based on the Chalearn Gesture Dataset [25]. IsoGD is a dynamic isolated gesture dataset collected by the Kinect sensor and contains two modalities of RGB and depth data. The dataset was completed by 21 human performers, including 249 types of gestures. Each modality of data contains 47,933 labeled videos. This includes 35,878 videos in the training set, 5784 videos in the validation set, and 6271 videos in the test set. The video samples in the IsoGD dataset have a resolution of 320×240, and the length of each sample varies from 9 to 405 frames.

Briareo dataset is a dynamic gesture dataset for hand gesture recognition tasks in the automotive context. This dataset was collected from an innovative point of view: the acquisition devices were placed in the central tunnel between the driver and the passenger seats, oriented towards the car ceiling. Several kinds of data were provided in this dataset, including RGB images, depth maps, infrared intensities, raw and rectified infrared images, and 3D hand joints. This dataset included 12 gesture classes which were performed by 40 subjects. Every subject performed each gesture 3 times, leading to a total of 120 collected sequences. Each sequence lasted at least 40 frames.

### 4.2. Implementation Details

Our experiments were conducted on a server with an Intel(R) Xeon(R) Gold 5115 CPU @ 2.40 GHz and two Tesla P40 GPUs. All model training and testing phases were based on the Pytorch framework.

Due to the different sample lengths in the IsoGD and Briareo datasets, in order to uniformly input the samples into the convolutional neural network, we uniformly processed the videos into 16 frames for IsoGD and 32 frames for Briareo, according to the method of equidistant sampling with random jitter. The I3D model we used was pre-trained on the Kinetics 400 dataset [26]. Each frame of the video sample was randomly cropped to 224×224 while training. During the inference phase, frames were center-cropped to the same 224×224 size. In addition, we used stochastic gradient descent to optimize the neural network parameters. The initial learning rate was set to 0.01, and the learning rate was multiplied by 0.1 after every 3 epochs. The momentum was set to 0.9, and the weight decay was set to 0.00001. The training phase lasted for 30 epochs.

### 4.3. Comparison with State-of-the-Art Methods on the IsoGD Dataset

Our method was compared with current state-of-the-art methods on the IsoGD dataset. Table 1 shows the comparison on IsoGD. To compare some of the methods of using single RGB/depth data, we tested the proposed method using different modality data. DA-3D indicated that the dual attention module was added on the basis of I3D, which was used for processing depth data. 

As shown in Table 1, our approach achieved the best performance whether using RGB data, depth data, or a fusion of RGB-D data. For the single modality of RGB data, our network with the multi-scale attention outperformed the second-best method by about 0.07%. The second-best method’s backbone is also the I3D, but it leveraged Network Architecture Search (NAS) for better feature extraction ability. It shows that even without a sophisticated network searching process, the network can still reach high accuracy with a comprehensive attention scheme. For depth data, the recognition accuracy of our network was about 1.06% higher than the second best network from Zhou et al. [27]. Note that the local attention module was not applicable, as the hand-in-depth data were not detected. Even so, the performance of our network was still considerable. With the multimodal fusion module, our method also achieved better performance for RGB-D data, which was about 1.53% improvement, compared with Zhou et al. [27].

**Table 1:** Comparison with state-of-the-art methods on IsoGD.

Method	Modality	Acc (%)
Wang et al. [28]	RGB	36.60
Li et al. [15]	RGB	37.28
Hu, Lin and Hsiu [29]	RGB	44.88
Miao et al. [14]	RGB	45.07
Duan et al. [16]	RGB	46.08
Zhang et al. [30]	RGB	51.31
Zhang et al. [31]	RGB	55.98
Zhu et al. [27]	RGB	57.42
Zhou et al. [1]	RGB	62.66
proposed	RGB	**62.73**
Wang et al. [28]	Depth	40.08
Miao et al. [14]	Depth	40.49
Li et al. [13]	Depth	48.44
Hu, Lin and Hsiu [29]	Depth	48.96
Zhang et al. [30]	Depth	49.81
Zhang et al. [31]	Depth	53.28
Zhu et al. [27]	Depth	54.18
Duan et al. [16]	Depth	54.95
Zhou et al. [1]	Depth	60.66
proposed (DA-3D)	Depth	**61.72**
Wang et al. [28]	RGB-D	44.80
Hu, Lin and Hsiu [29]	RGB-D	54.14
Zhang et al. [31]	RGB-D	55.29
Zhu et al. [27]	RGB-D	61.05
Zhou et al. [1]	RGB-D	66.62
proposed	RGB-D	**68.15**

### 4.4. Comparison with State-of-the-Art Methods on the Briareo Dataset

Table 2 shows the comparison of our method with other state-of-the-art methods on the Briareo dataset. Similar to Section 4.3, we tested our method with RGB data, along with depth data provided in Briareo. Therefore, in Table 2 we compared the methods based only these two modalities. 

From the single modality shown in Table 2, our method outperformed state-of-the-art methods by 0.7% for RGB data and 0.3% for depth data. For multimodal data, our method achieved 94.1%—the same accuracy as state-of-the-art methods. It can also be seen that, while we did not use complex network structures such as LSTM [32] and transformer [33], our results still sufficiently outperformed the state-of-the-art methods.

### 4.5. Ablation Studies

In this section, we verified the performance of our proposed method. We took the I3D network as the baseline and performed multimodal fusion, using RGB and depth modal data. To reflect the performance of the proposed modules, we gradually added components to the I3D network. Specifically, as shown in Table 3, it can be seen that the accuracy of the DA-3D network—the I3D network with dual spatiotemporal attention module that we proposed—was 0.42% higher for RGB data, and 0.15% higher in depth data than the I3D. This improvement can be attributed to the spatiotemporal contextual information captured by the dual spatiotemporal attention module, as well as the spatiotemporal channel contextual information. In addition, the accuracy of MSA-3D—the I3D network with dual spatiotemporal attention module and hand local attention module we proposed—was 1.45% higher than the I3D model, and 1.03% higher than the DA-3D model. It showed that the local attention module for the hand can reduce the influence of factors unrelated to gesture actions, and improve the model’s attention to hand actions.

### 4.6. Visual Analysis

**Figure 5:**
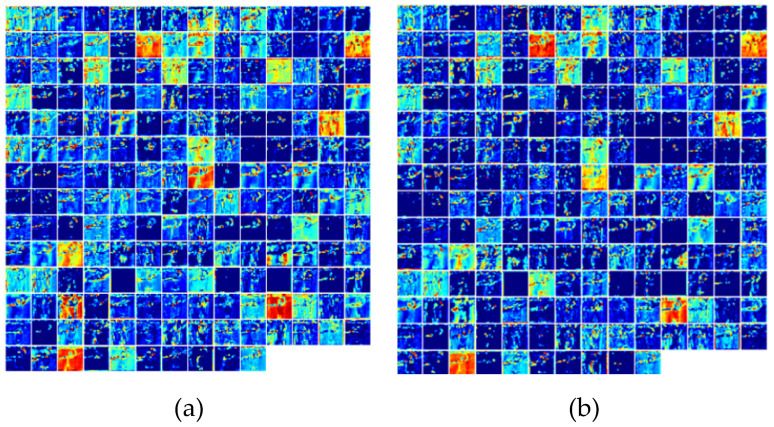
Comparison of channel feature map between DA-3D and MSA-3D: (**a**) features of each channel extracted after the third layer of DA-3D network; (**b**) features of each channel extracted after the third layer of MSA-3D network.

**Figure 6:**
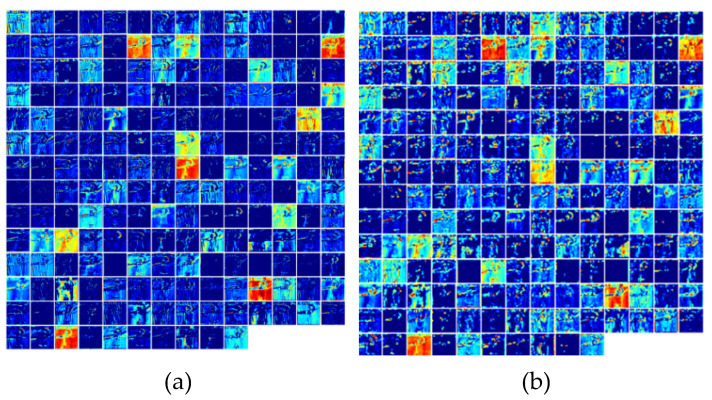
Comparison of channel feature map between I3D and MSA-3D: (**a**) features of each channel extracted after the third layer of I3D network; (**b**) features of each channel extracted after the third layer of MSA-3D network.

In order to intuitively show the effectiveness of the attention mechanism, this section visualized the channel features of the different models mentioned above. Figure 5a shows the extracted features of each channel after the third layer of DA-3D network processing with the dual attention mechanism. Compared with the MSA-3D, which added a local attention mechanism focusing on the hand (as shown in Figure 5b), the interference from the background and other irrelevant factors was significantly reduced. Figure 6a shows the extracted features of each channel after the third layer of the I3D network, without the attention mechanism. Compared with MSA-3D, which had a higher response to the gesture recognition task, was well as in the same channel (as shown in Figure 6b), the gesture features related to gesture recognition were more pronounced.

## 5. Conclusions

This paper proposed a local attention- and dual attention-based multimodal 3D Convolutional Network. The RGB and depth modal data provided in the IsoGD dataset and Briareo dataset, along with the hand videos generated by the RGB data, were used as input. The network used the I3D model based on dual spatiotemporal attention and local attention mechanisms to extract features of RGB data, and used the I3D model with dual spatiotemporal attention to extract depth data features. The extracted features were multiplied and fused element-wise as the final classification result. This method achieved 68.15% accuracy on the test set of IsoGD, which outperformed the baseline model I3D we used, as well as current state-of-the-art methods. This further illustrated the effectiveness of our model in capturing spatiotemporal context dependence and the full use of multimodal data.

## Figures and Tables

**Table 2:** Comparison with state-of-the-art methods in Briareo.

Method	Modality	Acc (%)
Manganaro et al. [32]	RGB	72.2%
Manganaro et al. [32]	Depth	76.0%
D’Eusanio et al. [33]	RGB	90.6%
D’Eusanio et al. [33]	Depth	92.4%
D’Eusanio et al. [33]	RGB-D	**94.1%**
proposed	RGB	91.3%
proposed (DA-3D) proposed	Depth	92.7%
	RGB-D	**94.1%**

**Table 3:** Accuracy of derivative models with RGB data.

Method	Acc (%)
I3D	61.28
DA-3D ^1^	61.70
MSA-3D ^2^	62.73

^1^ DA-3D means I3D network with dual spatiotemporal attention module. ^2^ MSA-3D means I3D network with dual spatiotemporal attention and hand local attention module.

## Data Availability

Not applicable.
